# RIT1 suppresses esophageal squamous cell carcinoma growth and metastasis and predicts good prognosis

**DOI:** 10.1038/s41419-018-0979-x

**Published:** 2018-10-22

**Authors:** Yan-Fen Feng, Yi-Yan Lei, Jia-Bin Lu, Shao-Yan Xi, Yu Zhang, Qi-Tao Huang, Qiu-Liang Wu, Fang Wang

**Affiliations:** 1 Department of Pathology, Sun Yat-sen University Cancer Center, 510060 Guangzhou, China; 2 Department of Thoracic Surgery, The First Affiliated Hospital of Sun Yat-sen University, 510060 Guangzhou, China; 3 Department of Molecular Diagnostics, Sun Yat-sen University Cancer Center, 510060 Guangzhou, China

## Abstract

Ras-like without CAAX1 (RIT1) protein is a member of Ras family, which plays critical roles in signaling pathways and cellular process regulation. However, the role of RIT1 in esophageal squamous cell carcinoma (ESCC) is unclear. In this study, we found that the expression of RIT1 is downregulated in ESCC compared to corresponding non-tumor tissues. The low-level expression of RIT1 was correlated with poorer prognosis. Then we showed that RIT1 inhibited proliferation, invasion, and migration of ESCC cells, and silencing RIT1 by shRNA promoted tumorigenicity and metastasis in nude mice. We further demonstrated that RIT1 inhibited the malignant behaviors of ESCC through inhibiting the PI3K/AKT and MAPK pathway and epithelial–mesenchymal transition in ESCC cells. Our study also revealed that RIT1 increased drug sensitivity to cisplatin (CDDP), and this function could be carried out through downregulating stemness of ESCC. In conclusion, our study indicates for the first time that RIT1 displays tumor-suppressing functions in ESCC, and these functions were carried out by inhibiting MAPK and PI3K/AKT signaling pathway, inhibiting EMT, and downregulating cancer stemness of ESCC cells.

## Introduction

Esophageal cancer is the eighth most common cancer in the world, with an estimated 456,000 incident cases and 400,200 deaths in the year 2012^1^. It has a distinct geographic distribution. Southern China is one of the districts with high incidence. Esophageal cancer is primarily composed of two histologic types: esophageal squamous cell carcinoma (ESCC) and esophageal adenocarcinoma (EA). ESCC is the predominate subtype, especially in Asian countries^2^. Because the clinical symptoms are obscure during early stage of the disease, many patients were diagnosed with advanced disease. Treatments for esophageal cancer include esophagectomy alone or combined with chemoradiotherapy or chemotherapy^3^. Although much progress has been made in treatment modalities, the outcome of treatment is still beyond satisfaction. The prognosis is inferior, and the overall 5-year survival rate is approximately 17%^4^. The factors affecting the prognosis include length of tumor, the number and ratio of involved lymph nodes, etc^5^.

Ras is a member of Ras super-family of small GTPase, which functions as binding switches of guanine nucleotide, and involve in many different kinds of cell functions, such as cell growth, differentiation, and apoptosis^6^. Ras family G-proteins transmits cellular signals to specific effectors, which results in the activation of diverse signaling pathways, including mitogen-activated protein kinase (MAPK) family protein kinases, phosphatidylinositol 3-kinase (PI3K)/AKT [protein kinase B (PKB)]^7^. It has been revealed that MAPK and PI3K/AKT signaling pathway activation correlate with many human cancers^8,9^. RIT1 (Ras-like-without-CAAX-1) is a member of Ras family, which possesses intrinsic GTP hydrolysis activity and is most highly homologous with members of Ras subfamily^10^. However, it has some unique biochemical properties and displays diverse and complicated biological functions. For example, RIT1 has been shown to play an important part in neuron survival following oxidative stress^11^, and it also contributed to dendritic cell retraction^12^. Studies also showed that RIT1 played a critical role in hepatocellular carcinoma, lung adenocarcinoma, myeloid malignancies, and endometrial carcinoma^13–16^. RIT1 was also considered to be a driver oncogene in a specific subset of lung adenocarcinoma^14^. Recent study revealed that expression of RIT1 correlated with poor prognosis in endometrial cancer^15^. However, the biological function of RIT1 in ESCC is still unclear. Herein we studied the role of RIT1 and its underlying regulatory mechanisms in ESCC.

## Results

### RIT1 was downregulated in ESCC and associated with poorer prognosis

**Fig. 1:**
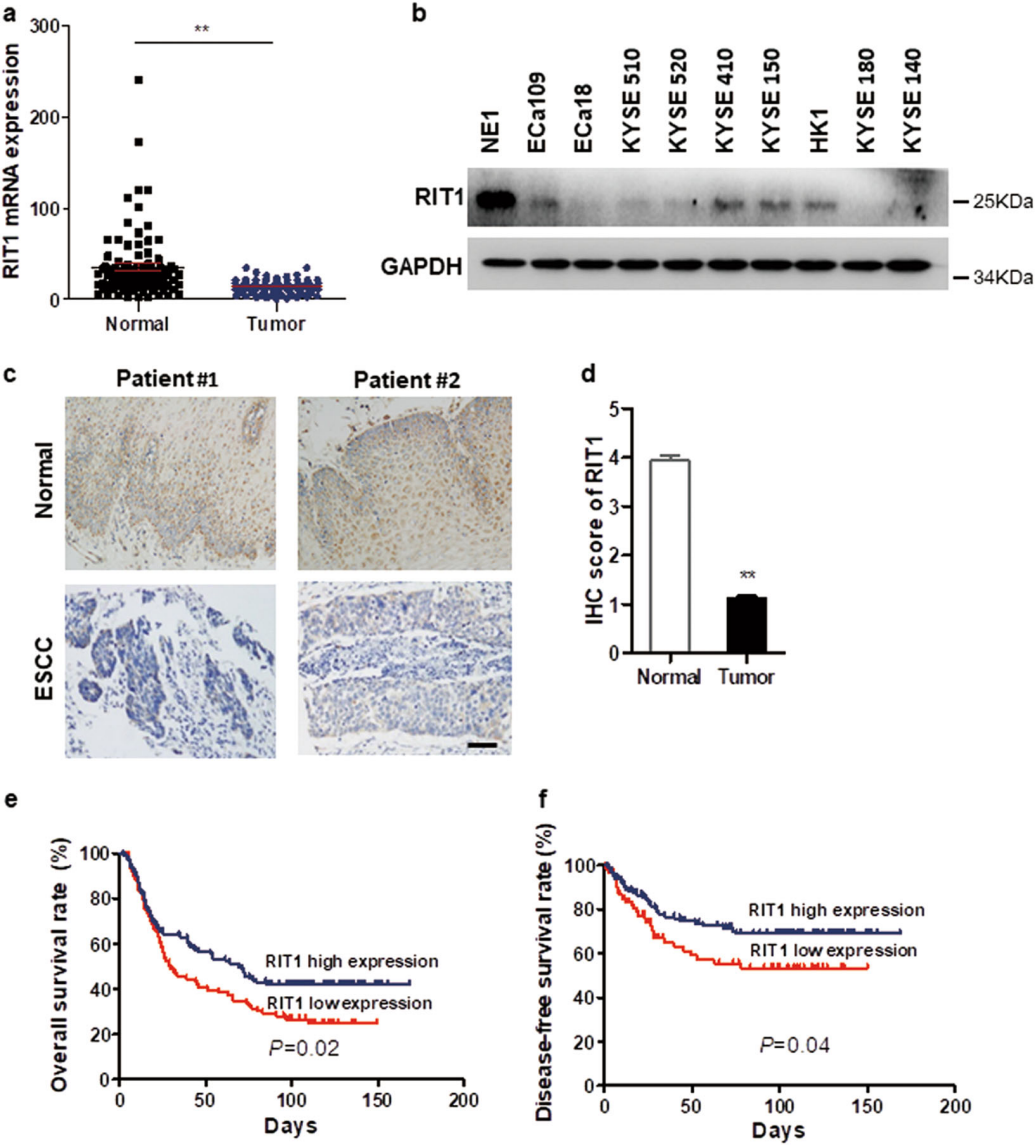
RIT1 is downregulated in ESCCs and is correlated with poor prognosis. **a** RIT1 expression was compared by qRT-PCR between tumor and corresponding non-tumor tissues in 96 ESCCs (**P < 0.01). **b** Expression of RIT1 was detected in all the tested ESCC cell lines by western blot analysis (WB) compared with an immortalized esophageal epithelial cell line NE1. **c**, **d** The expression scores were detected by immunohistochemistry (IHC) in 268 cases of ESCC tumor tissues compared with corresponding non-tumor tissues. **c** Representative images of RIT1 IHC staining in 2 pairs of ESCC cases (original magnification: ×200, calibration bar 25 μm). **d** Results are summarized as mean ± SEM (**P *<* 0.01). **e**, **f** Kaplan–Meier analysis showed the overall survival rate and disease-free survival rate of ESCC patients stratified by RIT1 expression

Expression of RIT1 was tested by quantitative real-time PCR (qRT-PCR) and compared between tumor and paired non-tumor tissues in 96 ESCC cases. The average fold change of RIT1 mRNA was significantly lower in ESCC tumor tissues than those in paired non-tumor tissues (13.7- vs. 23.6-fold changes) (Fig. 1a). Western blot (WB) analysis showed that the expression of RIT1 was lower in all the ESCC cell lines compared with the immortalized esophageal epithelial cell line NE1 (Fig. 1b). Expression of RIT1 was also investigated by immunohistochemistry (IHC) with a monoclonal RIT1 antibody using an ESCC tissue microarray containing 228 pairs of ESCC tumor and corresponding non-tumor tissues. The expression scores were significantly lower in tumor tissues (mean ± SEM: 3.295 ± 0.1345) than those in non-tumor tissues (mean ± SEM: 2.138 ± 0.1422) (Fig. 1c, d). The correlation of RIT1 expression with ESCC prognosis was analyzed statistically using IHC data from 228 informative ESCCs. The RIT1 expression level was considered high when the final scores were ≥median (score = 4) and low when the final scores were <median (score = 4). Kaplan–Meier analysis showed that the overall survival rate (OS) and disease-free survival rate (DFS) of ESCC patients with RIT1 low expression was significantly poorer (Fig. 1e, f). Multivariate analysis showed that RIT1 was an independent prognostic factor (Table 1).

**Table 1:** Univariate analysis and multivariate analysis of prognostic factors to predict OS

Prognostic parameter	Univariate analysis			Multivariate analysis		
	HR	95% CI	*P* value	HR	95% CI	*P* value
Expression of RIT1 (low vs. high)	1.449	1.04–2.019	**0.029**	1.473	1.053–2.061	**0.024**
Age	0.960	0.691–1.335	0.809	—	—	—
Gender	1.586	1.060–2.373	**0.025**	1.216	0.992–1.491	**0.060**
Lymph-vascular invasion	2.191	1.555–3.088	**0.000**	0.579	0.407–0.825	**0.002**
Perineural invasion	1.374	0.987–1.912	0.059	—	—	—
Stage	0.301	0.214–0.424	**0.000**	2.821	1.464–5.434	**0.002**
Histological differentiation	0.611	0.425–0.877	**0.008**	1.253	0.867–1.809	0.230
Lymph node metastasis	0.360	0.255–0.508	**0.000**	1.021	0.532–1.960	0.951

*HR*: Hazard ratio; *CI*: confidential interval

### RIT1 inhibited proliferation of ESCC cells

**Fig. 2:**
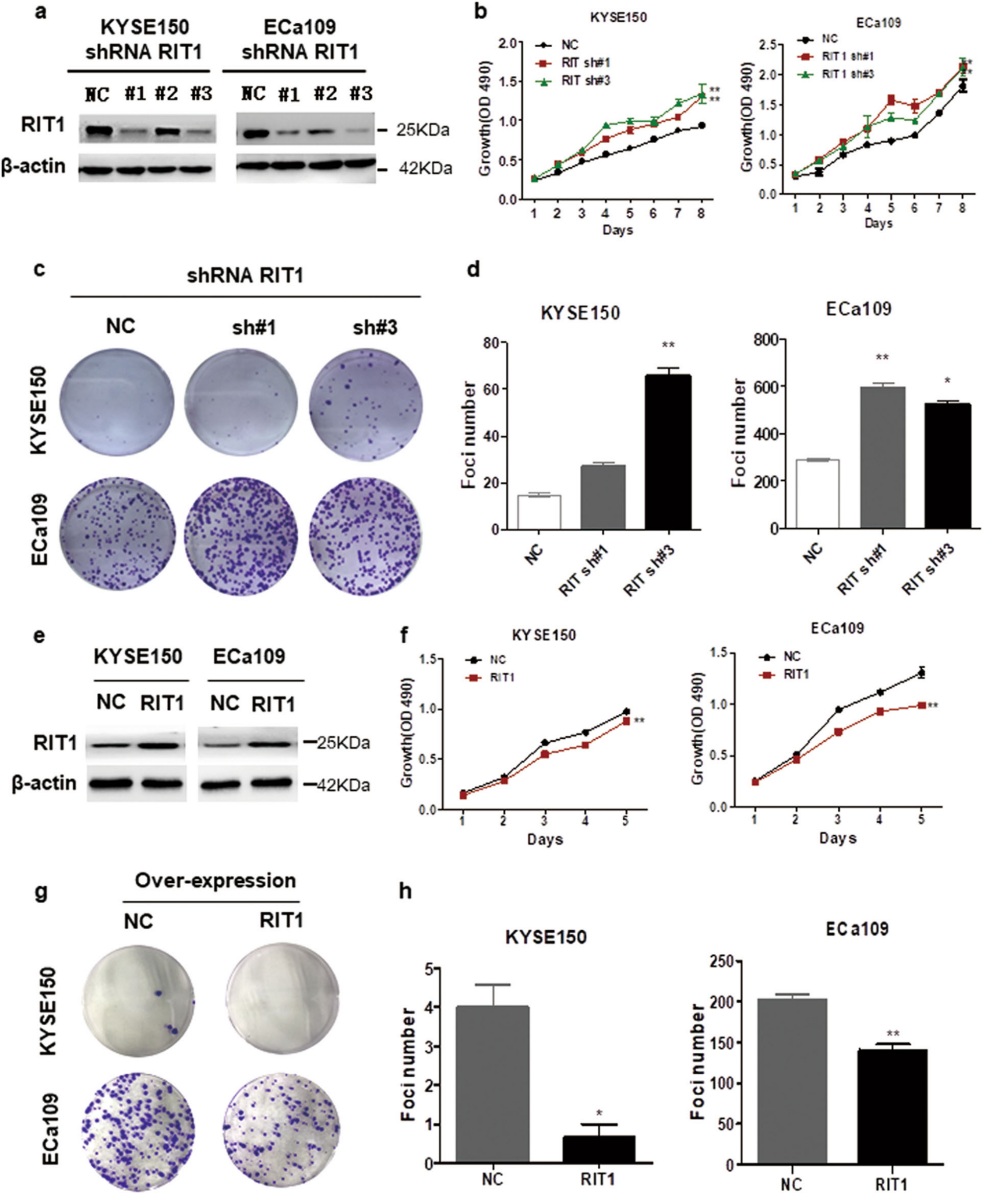
RIT1 inhibited proliferation of ESCC cells. **a**–**c** KYSE150 and ECa109 cells were transfected with shRNAs specifically targeting RIT1 (sh#1, sh#2, sh#3) or scrambled shRNA control (NC). **a** The effective knockdown of RIT1 in these ESCC cells was confirmed by western blot. **b** Cell growth rate of these RIT1 knockdown cells were detected by MTX and statistically analyzed. **c**, **d** Colony formation was detected by single-cell clone assay. **c** Representative image of clone formation are shown. **d** Results are statistically analyzed. **e**–**h** KYSE150 and ECa109 cells were transfected with RIT1 expression plasmid or mock control (NC). **e** Successful overexpression of RIT1 was confirmed by western blot. **f** Cell growth rate were detected by MTX and statistically analyzed. **g**, **h** Colony formation was detected by single-cell clone assay. **g** Representative image of colony formation are shown. **h** Results are statistically analyzed. The presented figures are representative data from at least three independent experiments. *P < 0.05, **P < 0.01 for statistical analysis of the indicated groups

Since RIT1 low expression correlated with worse prognosis in ESCC patients, we speculated that RIT1 might act as a suppressing regulation factor in ESCC, and RIT1 expression may inhibit tumor cell growth. Thus the effect of RIT1 on cell growth was investigated by silencing the expression of RIT1. We stably knocked down RIT1 expression in KYSE150 and ECa109 cells using lentiviral RIT1 short hairpin RNAs (shRNAs). A scrambled shRNA was used as a negative control (NC). The expression of the RIT1 in RIT1-knocked-down cells was tested by qRT-PCR (Supplemental Fig. 1a) and WB (Fig. 2a). MTS (3-(4,5-dimethylthiazol-2-yl)-5-(3-carboxymethoxyphenyl)-2-(4-sulfophenyl)-2H-tetrazolium) assay showed that cell growth rates in RIT1-knocked-down KYSE150 and ECa109 cells were significantly higher than those in the NC cells (Fig. 2b). Focus formation assay yielded higher number and larger colonies in the RIT1-knocked-down KYSE150 and ECa109 cells compared to the control cells (Fig. 2c, d). The effect of RIT1 overexpression on cell growth was also studied. RIT1-overexpressed plasmid or NC was transfected into KYSE150 and ECa109 cells and successful overexpression of RIT1 was confirmed by qRT-PCR (Supplemental Fig. 1b) and WB (Fig. 2e). MTS assay showed that cell growth rates in RIT1-overexpressed cells were significantly lower than those in control cells (Fig. 2f). Focus formation assay yielded lower number and smaller colonies in RIT1-overexpressed cells (Fig. 2g, h). Results altogether indicated that RIT1 inhibited proliferation of ESCC cells.

### RIT1 inhibited migration and invasion of ESCC cells

**Fig. 3:**
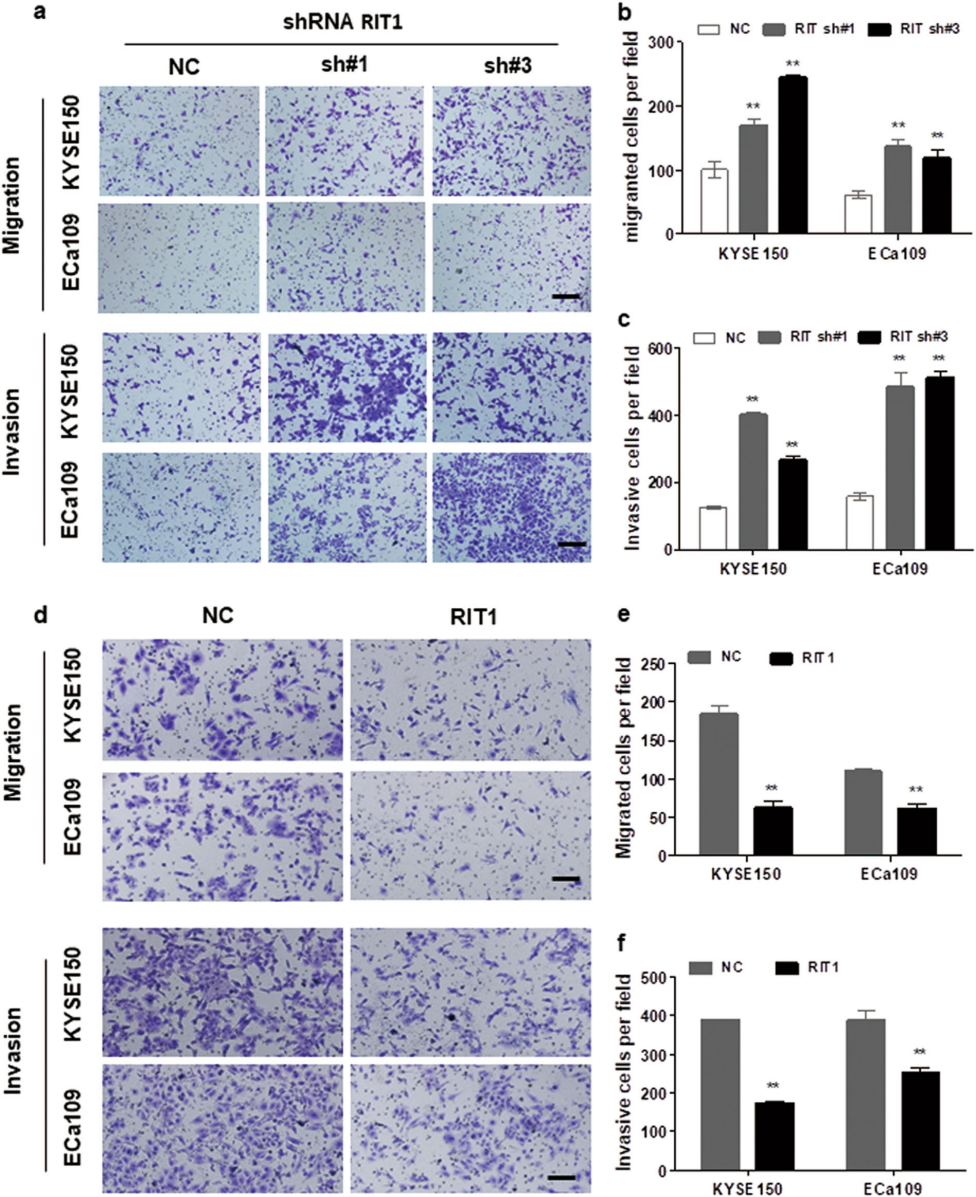
RIT1 inhibited invasion and migration of ESCC cells. **a**–**c** Transwell assay was performed to compare migration and invasion between cells treated with shRNAs and NC. **a** Representative images of migrated or invasive cells are shown (original magnification: ×100, calibration bar 50 μm). **b**, **c** Results are statistically analyzed. **d**–**f** Cell motilities tested by transwell assay were compared between cells treated with RIT1 expression plasmid and NC. **d** The representative images of migrated or invasive cells are shown (original magnification: ×100, calibration bar 50 μm). **e**, **f** Results are statistically analyzed. The presented figures are representative data from at least three independent experiments. **P < 0.01 for statistical analysis of the indicated groups

Previous study showed that RIT1 expression was closely correlated with clinical stage and vascular invasion in endometrial cancer^15^. To study the effect of RIT1 in ESCC cell migration and invasion, cell motility was studied after exogenous knockdown and overexpression of RIT1 in ESCC cells. Results showed that, when we knocked down RIT1 expression by two of its specific shRNAs, invasion and migration of ESCC cells were significantly increased (Fig. 3a–c), whereas exogenous overexpression of RIT1 significantly inhibited invasion and migration of the RIT1-overexpressed cells (Fig. 3d–f). These results indicated that RIT1 inhibited migration and invasion of ESCC cells.

### Silencing of RIT1 promoted tumorigenicity and metastasis in nude mice

**Fig. 4:**
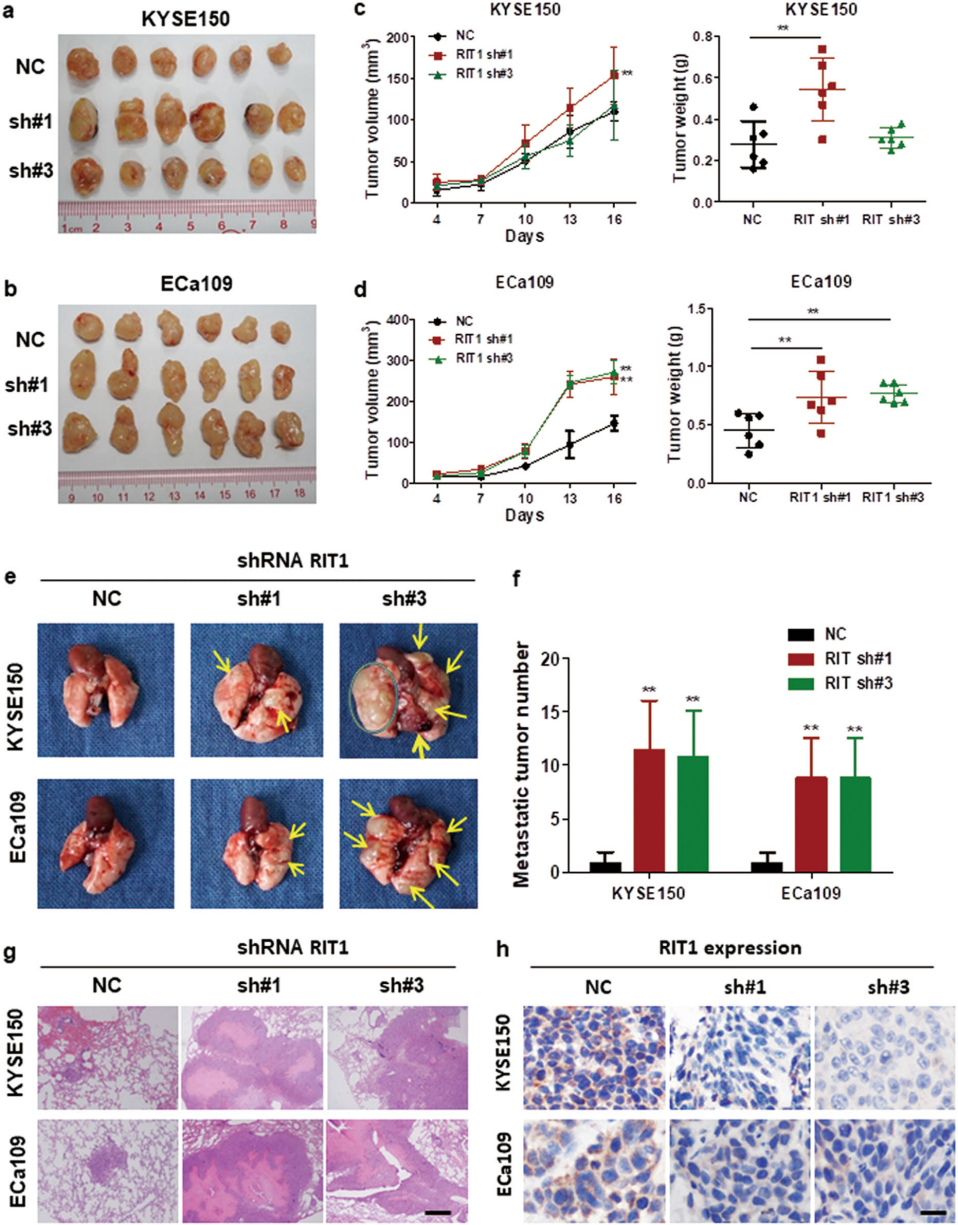
Silencing of RIT1 by shRNA promoted tumorigenicity and metastasis in nude mice. **a**, **b** Images of the xenograft tumors formed in nude mice injected with shRNA silencing of RIT1 (sh#1, sh#3) and scrambled shRNA control cells (NC). **c**, **d** Volume and weights of xenograft tumors are summarized. **e** Representative images of metastatic tumor nodules in the lung section of nude mice intravenously injected with shRNA silencing of RIT1 (sh#1, sh#3) and scrambled shRNA control cells (NC). **f** Number of metastatic tumor nodules in the lung are compared between nude mice injected with shRNA (sh#1, sh#3) and scrambled shRNA control cells (NC) and statistically analyzed. **g**, **h** Representative images of H&E and IHC expression of RIT1 in metastatic tumor nodules in the lung section of nude mice (original magnification: **g** ×40, calibration bar 125 μm, **h** ×200, calibration bar 25 μm). Data are presented as mean ± SEM. **P < 0.01 vs the control

To further confirm the oncogenic role of RIT1 in vivo, we established a xenograft tumor mouse model by subcutaneously injecting RIT1-knocked-down KYSE150 and ECa109 cells into the right dorsal flanks of a group of 8 nude mice, respectively. The same number of NC cells was injected into 8 nude mice as control. After 16 days, the mice were sacrificed. The size and weight of the xenograft tumors were measured. Results showed that tumors developed from RIT1-knocked-down cells were significantly larger and heavier than tumors from control cells (Fig. 4a–d). Hematoxylin and eosin (H&E) staining was used to confirm that the nodules developed in mice were tumors (Supplemental Fig. 2a). Results from the IHC staining confirmed that the Ki-67 expression in xenograft tumors that developed from the RIT1-knocked-down cells was significantly higher than tumors from control cells (Supplemental Figs. 2a, b). These results suggested that silencing of RIT1 inhibited ESCC tumor growth and proliferation. To evaluate the effects of RIT1 on tumor metastasis in vivo, three groups of six mice each were injected intravenously into the tail vein with RIT1-knocked-down KYSE150, ECa109, or NC cells, respectively. After 8 weeks, the mice were sacrificed, and the metastatic nodules in the lung surfaces were counted. A significantly larger number of metastatic nodules were induced at the surface of the lungs of mice injected with the RIT1-knocked-down cells than in those with the NC cells (Figs. 4e, f). H&E staining confirmed that the nodules on the surfaces of mice lungs were metastatic tumors (Fig. 4g). IHC staining was used to confirm that the expression of RIT1 in xenograft tumors that developed from the RIT1-knocked-down cells was lower than tumors from control cells (Fig. 4h). These results indicated that RIT1 inhibited invasion and metastasis of ESCC.

### RIT1 inhibited the MAPK and PI3K/AKT pathway and epithelial–mesenchymal transition (EMT) in ESCC cells

**Fig. 5:**
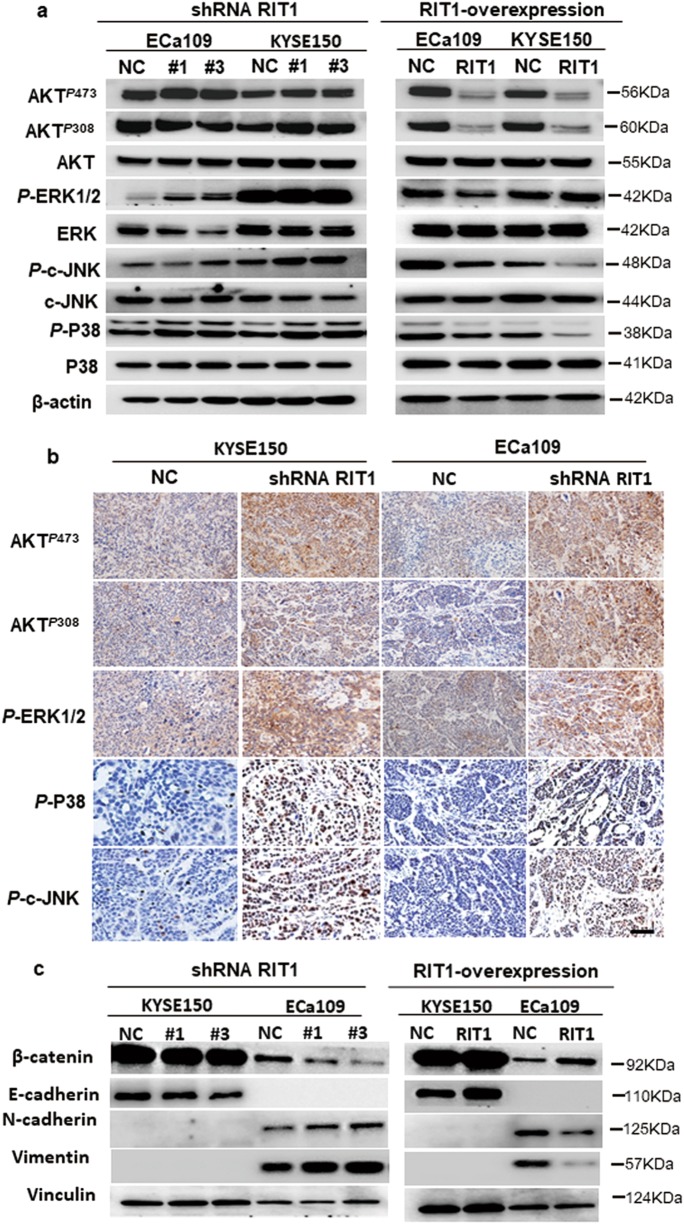
RIT1 inhibited the PI3K/AKT and MAPK pathways and epithelial–mesenchymal transition in ESCC cells. **a** Western blots comparing shRNA RIT1-silenced and RIT1-overexpressing cells with their respective control cells (NC) are seen in relative expression of AKT^*P*473^, AKT^*P*308^, AKT, *P*-ERK1/2, ERK, *P*-c-JNK, c-JNK, *P*-P38, P38, and β-actin was taken as control. **b** IHC comparing the expression of AKT^*P*473^, AKT^*P*308^, *P*-ERK1/2, *P*-c-JNK, and *P*-P38 in xenograft tumors developed from the shRNA RIT1-silenced cells (original magnification: ×200, calibration bar 25 μm). **c** Western blots comparing shRNA RIT1-silenced and RIT1-overexpressing cells and with their respective control cells (NC) are seen in relative expression of β-catenin, E-cadherin, N-cadherin, and vimentin. Vinculin was taken as control

As a Ras super family member, we speculated that, like other Ras GTPase, RIT1 played a role in the ESCC tumorigenesis via MAPK and PI3K/AKT pathways. To test the effect of RIT1 on these two pathways, WB was used to study the expression levels of phosphorylated extracellular signal–regulated kinase (ERK), c-Jun NH2-terminal kinase (c-JNK), P38, and AKT after exogenous knockdown and overexpression of RIT1 in ESCC cells. WB results showed that the phosphorylated ERK c-JNK, P38 (T180 and Y182), and AKT (S473 and T308) increased in RIT1-knocked-down cells and decreased in RIT1-overexpressed cells (Fig. 5a). IHC staining was used to confirm whether the expression of phosphorylated ERK, c-JNK, P38, and AKT in xenograft tumors that developed from the RIT1-knocked-down cells was higher than tumors from control cells (Fig. 5b). These results indicated that RIT1 inhibited both MAPK and AKT pathway in ESCC cells. Whether RIT1 inhibits tumor invasion and metastasis via inhibiting EMT has not been studied. The expression levels of EMT markers and EMT-related transcription factors were investigated to analyze the effect of RIT1 on EMT. qRT-PCR results showed significantly decreased expression of epithelial markers E-cadherin and β-catenin, whereas increased expression of mesenchymal marker Vimentin and Fibronectin in cells with knocked-down RIT1 compared to the control cells (Supplemental Fig. 3a). On the other hand, qRT-PCR revealed significantly increased expression of epithelial markers E-cadherin, a-catenin and β-catenin, whereas decreased expression of mesenchymal marker Vimentin and Fibronectin in RIT1-overexpressed cells (Supplemental Fig. 3b). WB results were in line with the qRT-PCR results (Fig. 5c). Taken all together, our results indicated that RIT1 inhibited tumor tumorigenesis and metastasis via inhibiting MAPK and PI3K/AKT pathways and EMT in ESCC, respectively.

### RIT1 increased drug sensitivity to CDDP and downregulating stemness of ESCC cells

**Fig. 6:**
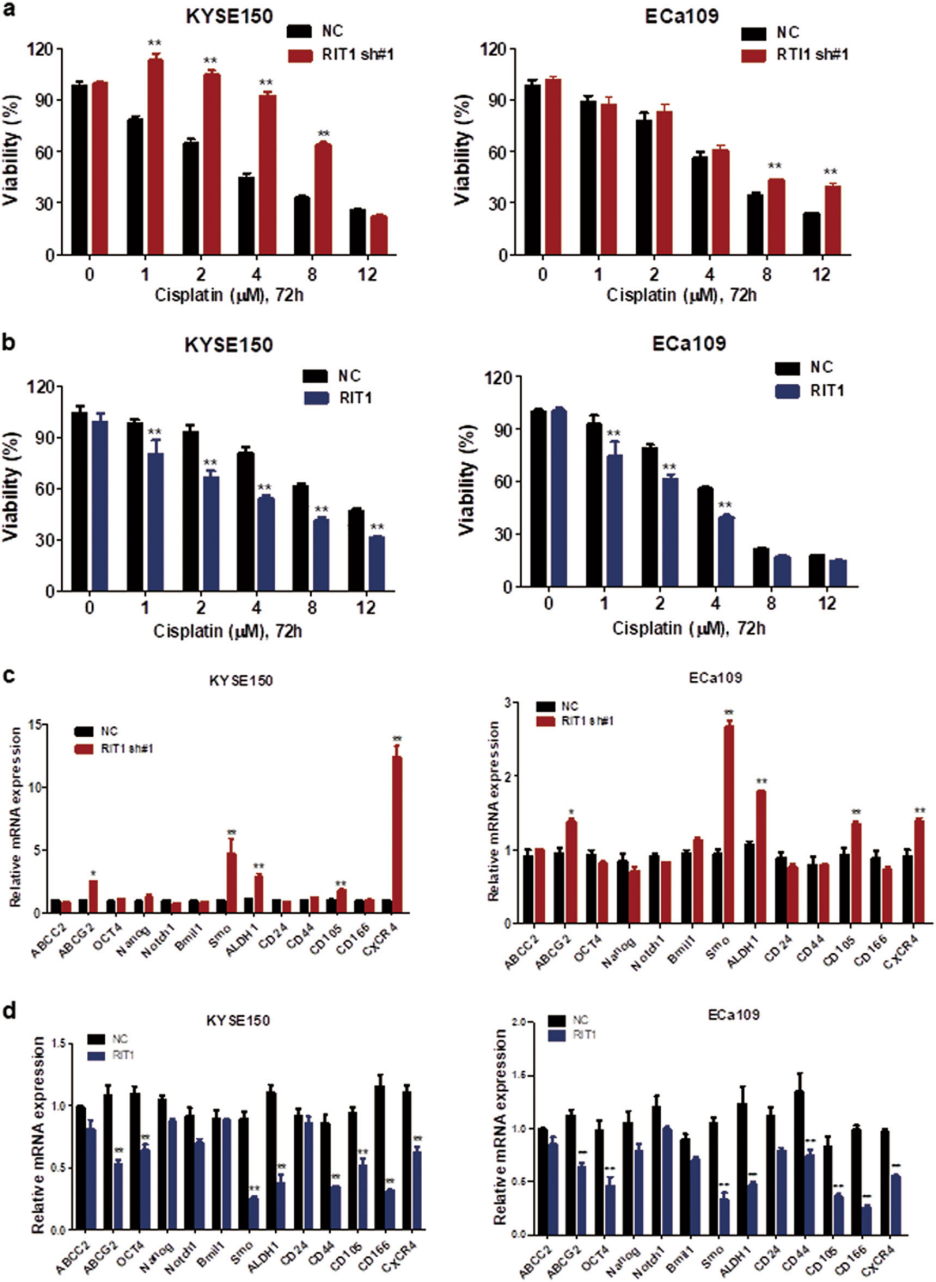
RIT1 increased drug sensitivity to Cisplatin and downregulating the stemness of ESCC cells. **a**, **b** The viability of KYSE150 and ECa109 cells treated with Cisplatin (CDDP) at the indicated concentrations was detected with MTS assays. Results were compared between shRNA RIT1-silenced (**a**) and RIT1-overexpressing cells (**b**) with their respective control cells (NC). **c**, **d** Relative expression of multiple drug-resistant transporter genes (ABCC2, ABCG2), stemness-associated genes (OCT-4, Nanog, Notch-1, Bmi-1, and Smo), and surface antigens associated with cancer stem cells (CD24, CD44, CD105, CD166, and CXCR4) were compared by qPCR between RIT1-silenced and control cells (**c**) or RIT1-overexpressing and control cells (**d**). Data are presented as the mean ± SEM. *P < 0.05 or **P < 0.01 vs the control

To test the effect of RIT1 on drug resistance, drug sensitivity study was performed. Results showed that, after treatment with CDDP for 48 h, cell viability significantly increased in KYSE150 and ECa109 cells with knockdown of RIT1 (Fig. 6a), whereas significantly decreased in KYSE150 and ECa109 cells with overexpressing RIT1 (Fig. 6b), which indicated that RIT1 increased drug sensitivity to CDDP in ESCC cells. Since stemness of cancer has been indicated to be associated with drug resistance, we further studied whether RIT1 expression affects the stemness of ESCC. We used RT-PCR to study the expression of stemness-associated genes and stem cell-related surface marker. Results showed that, in ESCC cells with exogenous knockdown of RIT1, multiple drug-resistant transporter gene ABCG2, stemness-associated gene Smo and ALDH1, and cancer stem cell-related surface marker CD105 and CXCR4 were significantly upregulated (Fig. 6c), whereas in cells with exogenous overexpression of RIT1, multiple drug-resistant transporter gene ABCG2, stemness associated genes OCT4, smo, and ALDH1, as well as stem cell-related surface markers CD44, CD105, CD166, and CXCR4 were significantly downregulated (Fig. 6d). Together, we showed that RIT1 increased drug sensitivity to CDDP, and this function could be partially due to inhibiting stemness of ESCC cells.

## Discussion

Previous studies showed that RIT1 was overexpressed in about 25% of patients with hepatocellular carcinoma, due to amplification and occasionally mutation^13^. Next-generation sequencing revealed activating mutations and locus amplifications of RIT1 in subgroup of patients with myeloid neoplasms^16^. Somatic mutations were found in RIT1 in ~2% lung adenocarcinoma cases, and RIT1 was identified as a driver oncogene in a specific subset of lung adenocarcinoma^14^. Data from The Cancer Genome Atlas also show that RIT1 were overexpressed in pancreatic carcinoma, lung carcinoma, and tongue squamous cell carcinoma tissues. Recent report showed that RIT1 was overexpressed in endometrial carcinoma and correlated with poorer prognosis^15^. All these data support that RIT1 displayed an oncogenic function in those tumors. However, our study showed reverse results. We found that RIT1 was downregulated in ESCC tissues compared to adjacent normal esophageal epithelial tissues, and the low expression of RIT1 correlated with poorer prognosis. These results preliminarily revealed that RIT1 displays tumor-suppressing function in ESCC, which is different from all the previous studies.

To profoundly study the function of RIT1 in ESCC and its underlying mechanisms, a set of in vivo and in vitro assays were carried out. Results showed that RIT1 inhibited tumor growth and proliferation. We further demonstrated that RIT1 realized these functions by inhibiting MAPK and PI3K/AKT signaling pathway in ESCC. MAPK and PI3K/AKT pathways are important in regulating cell growth and also play critical role in tumorigenesis of some human cancers. MAPK cascades are key signaling pathways involved in the regulation of normal cell proliferation, survival, and differentiation. The mammalian MAPK family consists of ERK, c-JNK, and p38^17^. Aberrant regulation of MAPK cascades contributes to cancer and other human diseases^18,19^. The ERK pathway is the best studied of the mammalian MAPK pathways, which mainly associated with cell proliferation, and is deregulated in approximately one third of all human cancers^17^. The ERK signaling pathway is often upregulated in many tumors^8^. Although JNKs are primarily attributed to proapoptotic cell death or tumor suppression in response to a variety of stress, inflammatory, or oncogenic signals, emerging evidence suggests that JNKs, especially JNK1, play a role in the malignant transformation of cells and in tumorigenesis^20,21^. p38‑MAPK signaling has been implicated in the regulation of processes that lead to the development and progression of a variety of cancer types^22,23^. Furthermore, in certain other tumor types, the inhibition of p38 enhances sensitivity to chemotherapy^24^, suggesting that p38 may serve as an oncogene in cancer progression. In AKT pathway, AKT is a downstream target of the PI3K and plays an important role in cancer cell survival, cell cycle entry, and glucose metabolism^25^. Phosphorylation of AKT promotes tumorigenesis via several oncogenic events, including apoptosis and cell proliferation^9^.

Our study also found that RIT1 inhibited tumor invasion and metastasis. We further demonstrated that this function was carried out by inhibiting EMT. In recent years, EMT shows more and more important role in epithelial tumor invasion and metastasis. EMT is regulated by a series of complicated signaling pathways^26,27^, and its process is accompanied by profound changes in cell characteristics, which enable the epithelial cells to detach from tight junctions, change their shape and polarity, delaminate, and migrate^28,29^. Downregulation of E-cadherin, aberrant location of β-catenin, and nuclear expression of Vimentin are some of its hallmarks^28,29^. In the progression of epithelial tumors, tumor cells acquire phenotypes of invasion and motivation and then invade to adjacent tissues or metastasize, which contributes to 90% of patient death^30^. EMT is also linked to cancer stem cell (CSC) generation and maintenance. CSCs are subpopulation of cells having self-renewal and expanding capability, thus contributing to tumor growth, metastasis, and resistance to conventional therapies^31^.

To investigate the potential clinical treatment relevant of RIT1, we used drug sensitivity assay to test whether RIT1 is associated with chemoresistance of ESCC. Results showed that RIT1 increased drug sensitivity to CDDP. Chemoresistance is a major cause of treatment failure. Chemoresistance might result from the combined activation of many cellular processes involved in EMT and may be related to acquisition of stem-like features by cancer cells^30^. Activation of EMT has been associated with chemoresistance in different tumor types. Enrichment of cells expressing mesenchymal markers has been detected in breast, colorectal, and non-small lung cancers upon chemotherapeutic treatments^32–34^. Recent studies have suggested CSC as a main player for chemoresistance against a variety of drugs in many types of cancer, including gliomas and glioblastoma, breast, and colorectal cancers^35–37^. Thus the acquisition of EMT and cancer stem cell properties is an important factor in tumor progression, relapse, and treatment resistance.

In conclusion, the present data show that RIT1 was downregulated in ESCC and significantly associated with poorer prognosis. RIT1 functions as a tumor suppressor in ESCC. This function could be carried out by inhibiting MAPK and PI3K/AKT signaling pathway, EMT, and cancer stemness. To the best of our knowledge, this is the first study about the role of RIT1 in ESCC and also the first study revealing that RIT1 displayed tumor-suppressing function. But how is RIT1 activated, why is RIT1 harboring a distinct function, and how does it affect the pathways to fulfill the function in ESCC is still needed to be further studied.

## Materials and methods

### Tissue specimens

Primary tumor specimens of ESCC and paired non-cancerous tissues were taken from 96 patients who underwent esophagectomy at the Sun Yat-sen University Cancer Center (SYSUCC) (Guangzhou, China) from March 2012 to March 2013. All tissue samples were immediately quick-frozen with liquid nitrogen and preserved at −80 ℃ with RNAlater. All the patients did not receive neoadjuvant chemoradiotherapy. Ethics Committee of Sun Yat-sen University approved this study.

### Immunohistochemistry

A tissue array that contained 228 cases of ESCC were used for the study. Each case included two cancerous tissues and one matched pair para-cancerous normal tissue. All patients were treated at the Department of Thoracic Surgery of SYCUCC (Guangzhou, China) between January 2000 and December 2007, along with the available clinicopathological information. IHC was performed following the standard protocol described previously^38^. The slides were blocked and then primary antibodies [anti-RIT1 (1:800), anti-Ki-67 (1:500), anti- AKT^*P*473^ (1:100), AKT^*P*308^ (1:100), anti-*P*-ERK1/2 ^*p*T202/Y204^ (1:200), anti-c-JNK (1:100), *P*-c-JNK (1:100), P38 (1:100), and anti-*P*-P38 ^*P*T180/Y182^ (1:100)] were applied and incubated at a temperature of 4 °C overnight. Biotinylated goat anti-rabbit immunoglobulin were then applied on the tissue sections and incubated for 30 min at a concentration of 1:75 at 37 °C. Finally, tissue sections were developed with diaminobenzidine. Two independent pathologists who were blinded to the patients’ clinical characteristics and xenograft tissues assessed the IHC staining based on the percentage of positively stained tumor cells. The stained sections were evaluated in five representative fields from each section and the positively stained tumor cells were analyzed to determine the IHC scores. The proportion of RIT1-positive cells varied from 0 to 100% (the scores of the extent of positive cells in a microscopic field of view: <25% scores 0; 25–50% scores 1; 50–75% scores 2; 75–100% scores 3), and staining intensity varied from weak to strong. A final score was then calculated by adding these two scores. The Research Ethics Committee of SYSUCC approved this study.

### Cell culture

ESCC cell lines KYSE150, KYSE520, KYSE410, KYSE140, KYSE180, and HK1 were supplied by Deutsche Sammlung von Mikroorganismen und Zellkulturen (DSMZ, Braunschweig, Germany), while ESCC cell lines ECa109, ECa18, and immortalized esophageal epithelia cell line NE1 were graciously provided by Professor Libing Son (SYCUCC). ESCC cells were maintained in Dulbecco’s modified Eagle medium (Invitrogen, Carlsbad, CA, USA). NE1 cells were maintained in 1:1 mixed serum-free medium containing growth supplement keratinocytes and EpiLife medium containing 60 μM calcium (Invitrogen, Carlsbad, CA, USA). We authenticated all cell lines before use according to STR fingerprinting at the Medicine Lab of Forensic Medicine Department of SYSUCC (Guangzhou, China).

### Antibodies and reagents

CDDP was supplied by Selleck Chemicals (Houston, USA). Primary antibodies against the following proteins were used in this study: RIT1, AKT^*P*473^, AKT^*P*308^, AKT, β-actin, glyceraldehyde 3-phosphate dehydrogenase (GADPH), E-cadherin, Vimentin, N-cadherin (Cell Signaling Technology, Beverly, MA, USA); and *P*-ERK1/2^*P*T202/Y204^, ERK, c-JNK, P-c-JNK, P38, *P*-P38 ^*P*T180/Y182^, Vinculin, and Ki-67 (Abcam, Cambridge, MA, USA).

### Establishment of RIT1-knockdown and RIT1-overexpression cells

Lentiviral containing shRNAs targeting RIT1 was supplied by GenePharma (GenePharma Co., Ltd, Shanghai, China) and transfected into KYSE150 and ECa109 cells following the instructions of the manufacturer. And stable cell lines were selected with puromycin as previously described. The targets for G6PD shRNA were gctggacaggcagagtttaca (for RIT1 sh #1) and gctgcataccgctactatatt (for RIT1 sh #3). Cells transfected with scrambled shRNA were taken as a knockdown cell NC. RIT1 plasmid or NC was purchased from Obio Technology (Shanghai). Cells (KYSE150 and ECa109) were transfected with 5 μg plasmid using Lipofectamine 3000 and P3000 reagent (Invitrogen, Carlsbad, CA, USA) following the instructions of the manufacturer.

### RNA extraction and qRT-PCR

Trizol Reagent and Superscript III Reverse Transcriptase (Invitrogen, Carlsbad, CA, USA) were used to extract total RNA and perform reverse transcription, respectively. ABI PRISM 7900 Sequence Detector and SYBR Green PCR Kit (Applied Biosystems, Carlsbad, CA, USA) were used to perform qRT-PCR to detect the quantity of cDNA. SDS 2.3 software (Applied Biosystems, Foster City, CA, USA) was used to quantify and analyze the relative expression levels. GADPH was used as an endogenous reference. The experiments were repeated three times, and then the relative expression of genes was analyzed. In the Supporting Materials, we have listed the primer sequences.

### WB analysis

Whole-cell lysates were prepared and quantified according to standard protocols and then separated by sodium dodecyl sulfate-polyacrylamide gel electrophoresis (SDS-PAGE) and electrophoretically transferred to nitrocellulose membranes. The membranes were blocked in blocking buffer and incubated with monoclonal antibodies. The membranes were incubated with peroxidase-conjugated anti-rabbit secondary antibodies. To ensure equal loading, membranes were probed with an internal control antibody (GADPH, β-actin, Vinculin). In the Supporting Materials, we have listed the antibodies’ information.

### Cell proliferation assays

Cell proliferation was evaluated by using the MTS assay (Promega, Madison, WI, USA) following the instructions of the manufacturer. Briefly, we seeded about 1500 cells in 200 µL media into 96-well plates, and then cultured till the indicated days. Then we added 20 μL MTS solution in the plates incubated for an extra 2 h. The optical density of each well was measured with an enzymatic reader at 490 nm (Thermo Scientific, Waltham, MA, USA). We repeated the experiments at least three times and expressed the data as mean ± SEM.

### Focus formation assays

For focus formation assay, we seeded 500 cells in every 6-well plate. After 10 days of culture, crystal violet staining was used to count the cell colonies. We repeated the experiments at least three times and expressed the data as mean ± SEM.

### Transwell migration and invasion assays

Transwell chambers with or without matrix gel inserts for 24-well plates were purchased from Corning (Corning, NY, USA). In brief, we added 200 μL of medium without fetal bovine serum (FBS) containing 1 × 10^5^ cells in the upper chamber. And 600 μL of medium with 80% FBS in the lower chamber was used as a chemoattractant. Then we incubated the cells at a temperature of 37 °C for 24 h and removed cells remaining on the upper chamber surface with a cotton swab. Cells that migrated into the bottom of the filters were counted in five photographed fields after fixation with 4% formaldehyde and staining with 0.5% crystal violet.

### In vivo assays

Animal protocols were approved by the Institutional Animal Care and Use Committee of the Sun Yat-sen University. Nude mice (4–5 week old) were raised in an environment free of pathogen at the experimental animal center of the Sun Yat-sen University. Xenograft tumor growth models were established by subcutaneous injection of RIT1-knocked-down cells (RIT1 sh #1 and sh #3) and NC cells (2 × 10^6^ cells) into the right dorsal flank. Tumor growth in the nude mice was observed for 16 days. Tumor volume (*V*, cm^3^) was evaluated based on tumor length (*L*) and width (*W*) with the following formula: *V* = 1/2 × *L* × *W*^2^. In order to test how RIT1 affect tumor metastasis, we established metastatic tumor model by giving intravenous tail vein injections of 1 × 10^5^ RIT1-knocked-down cells (RIT1 sh #1 and sh #3) to two groups of mice. After 8 weeks, the mice were sacrificed, and the tumor nodules formed on the lung surfaces were counted. For better visualization, the lungs were placed beneath a dissecting microscope at ×10 magnification to count the lung foci. Each foci should appear as a circular, brown obtrusion on the surface of the lungs. For consistency between mice, the number of foci on the surface of a lobe was counted, and that lobe was then inverted. The procedure was carried out for each of the four lobes individually^39^.The tumors were embedded in paraffin for further study. All animal studies were conducted with the approval of the Sun Yat-sen University Institutional Animal Care and Use Committee.

### Drug sensitivity assays

We seeded KYSE150 and ECa109 cells with RIT1-knocked-down cells (RIT1 sh) in 96-well plates at a density of 1.5 × 10^3^ cells per well and gave treatment with cisplatin (CDDP) at different concentrations for 72 h. Cell viability was determined by MTS Cell Proliferation Assay (Promega, Madison, WI, USA). The experiments were repeated for three times.

### Statistical analysis

All analyses were performed by using the Graphpad prism 6 software (San Diego, CA, USA). One-way analysis of variance and Newman–Keuls multiple comparison tests were used for the comparison of the significant differences between more than two groups. Unpaired *t* test was used to assess the RIT1 expression between tumor tissues and corresponding non-tumor tissues. Survival was analyzed with Kaplan–Meier survival curves. The Cox proportional hazards regression model was used to identify independent prognostic factors. Data are presented as the mean ± SEM, *P < 0.05, **P < 0.01. A *P* value <0.05 was considered statistically significant.

## Electronic supplementary material

Supporting materials

Supplementary figure legends

Supplemental Figure 1

Supplemental Figure 2

Supplemental Figure 3
