# Supplementary material for: RIT1 suppresses esophageal squamous cell carcinoma growth and metastasis and predicts good prognosis
Source: Cell Death Dis. 2018 Oct 22;9(11):1085. doi: 10.1038/s41419-018-0979-x (PMC6197279; doi:10.1038/s41419-018-0979-x)
Supplement: Supplementary file 1 — Supporting materials [file 41419_2018_979_MOESM1_ESM.docx]

**Supporting materials**

1. **Sequencing of shRNA:**

sh#1：GCTGGACAGGCAGAGTTTACA

sh#2：GGATCGTCGAAGTTTCCATGA

sh#3：GCTGCATACCGCTACTATATT

1. **Primer sequencing of markers tested in the study**

**RIT1**

Forward Primer: TTCATCAGCCACCGATTCCC

Reverse Primer: GCAGGCTCATCATCAATACGGA

**E-cadherin**

Forward Primer: TGCCCAGAAAATGAAAAAGG

Reverse Primer: GTGTATGTGGCAATGCGTTC

**a-catenin**

Forward Primer: AGCGAATTGTGGCAGAGTGT

Reverse Primer: GTCTACGCAAGTCCCTGGTC

**β-catenin**

Forward Primer: ACAACTGTTTTGAAAATCCA

Reverse Primer: CGAGTCATTGCATACTGTCC

**Vimentin**

Forward Primer: GAGAACTTTGCCGTTGAAGC

Reverse Primer: GCTTCCTGTAGGTGGCAATC

**Fibronctin**

Forward Primer: CAGTGGGAGACCTCGAGAAG

Reverse Primer: TCCCTCGGAACATCAGAAAC

**Slug**

Forward Primer: GGGGAGAAGCCTTTTTCTTG

Reverse Primer: TCCTCATGTTTGTGCAGGAG

**Snail**

Forward Primer: CCTCCCTGTCAGATGAGGAC

Reverse Primer: CCAGGCTGAGGTATTCCTTG

**ABCC2**

Forward Primer: ATGCAGCCTCCATAACCATGA

Reverse Primer: CTTCGTCTTCCTTCAGGCTATTCA

**ABCG2**

Forward Primer: TCATCAGCCTCGATATTCCATCT

Reverse Primer: GGCCCGTGGAACATAAGTCTT

**OCT4**

Forward Primer: CTTGCTGCAGAAGTGGGTGGAGGAA

Reverse Primer: CTGCAGTGTGGGTTTCGGGCA

**Nanog**

Forward Primer: AATACCTCAGCCTCCAGCAGATG

Reverse Primer: TGCGTCACACCATTGCTATTCTTC

**Bmi1**

Forward Primer: TGGAGAAGGAATGGTCCACTTC

Reverse Primer: GTGAGGAAACTGTGGATGAGGA

**Notch1**

Forward Primer: CCTGAGGGCTTCAAAGTGTC

Reverse Primer: GGAACTTCTTGGTCTCCAG

**Smo**

Forward Primer: TGGTCACTCCCCTTTGTCCTCAC

Reverse Primer: GCACGGTATCGGTAGTTCTTGTAGC

**CD24**

Forward Primer: TGAAGAACATGTGAGAGGTTTGAC

Reverse Primer: GAAAACTGAATCTCCATTCCACAA

**CD44**

Forward Primer: TTGCAGTCAACAGTCGAAGAAG

Reverse Primer: CCTTGTTCACCAAATGCACCA

**CD105**

Forward Primer: CGCCAACCACAACATGCAG

Reverse Primer: GCTCCACGAAGGATGCCAC

**CD166**

Forward Primer: TCCTGCCGTCTGCTCTTCT

Reverse Primer: TTCTGAGGTACGTCAAGTCGG

**CXCR4**

Forward Primer: ACTACACCGAGGAAATGGGCT

Reverse Primer: TTCTTCACGGAAACAGGGTT

1. **Antibodies:**

The dilution of RIT1, E-cadherin, β-catenin, N-cadherin, Vimentin, AKT, *P*-AKT^S473^ *, P*-AKT^T308^ ,ERK, *P*-ERK1/2, *P*-c-JNK, c-JNK, *P*-P38 *^P^*^T180/Y182^ and P38 used for western blot was 1：1000, while the dilution of β-actin, Vinculin and GADPH was 1：5000
