# Supplementary material for: RIT1 suppresses esophageal squamous cell carcinoma growth and metastasis and predicts good prognosis
Source: Cell Death Dis. 2018 Oct 22;9(11):1085. doi: 10.1038/s41419-018-0979-x (PMC6197279; doi:10.1038/s41419-018-0979-x)
Supplement: Supplementary file 2 — Supplementary figure legends [file 41419_2018_979_MOESM2_ESM.docx]

Supplemental Figure 1. The effective knockdown or overexpressing of RIT1 in ESCC cells was confirmed by qPCR. (a)Relative expressions of RIT1 were compared by qPCR between shRNA RIT1-silenced KYSE150 and ECa109 with their respective control cells (NC). (b) Relative expressions of RIT1 were compared by qPCR between RIT1-overexpressing cells (RIT1) and their respective control cells (NC). Data are presented as the mean ± SEM. ***P*<0.01 versus the control.

Supplemental Figure 2. The effect of RIT1 on tumor cell proliferation of xenograft tumor in nude mice. (a) Representative images of H&E and IHC expression of Ki-67 in tumor cells of the xenograft tumor section of nude mice (original magnification: ×200, calibration bar 25 μm). Data are presented as the mean ± SEM. ***P*<0.01 versus the control.

Supplemental Figure 3. The effect of RIT1 on EMT. Relative expressions of E-cadherin, α-catenin, β-catenin, Vimentin, Fibronectin and Slug in KYSE150 and ECa109 cells were compared by qPCR between shRNA RIT1-silenced (a) and RIT1-overexpressing cells （b）and with their respective control cells (NC). Data are presented as the mean ± SEM. **P*<0.05 or ***P*<0.01 versus the control.
